# Hereditary Breast and Ovarian Cancer Service in Sparsely Populated Western Pomerania

**DOI:** 10.3390/healthcare10102021

**Published:** 2022-10-13

**Authors:** Ute Felbor, Robin Bülow, Rita K. Schmutzler, Matthias Rath

**Affiliations:** 1 Department of Human Genetics, University Medicine Greifswald, Interfaculty Institute of Genetics and Functional Genomics, University of Greifswald, 17475 Greifswald, Germany; 2 Institute of Diagnostic Radiology and Neuroradiology, University Medicine Greifswald, 17475 Greifswald, Germany; 3 Center for Familial Breast and Ovarian Cancer, Center for Integrated Oncology (CIO), Faculty of Medicine, University Hospital of Cologne, 50937 Cologne, Germany

**Keywords:** hereditary breast and ovarian cancer, genetic counseling, diagnostic and predictive genetic analyses, risk-adapted prevention, rural area

## Abstract

The German Consortium Hereditary Breast and Ovarian Cancer (GC-HBOC) consists of 23 academic centers striving to provide high-quality regional care for affected individuals and healthy at-risk family members. According to the standard operating procedures defined by the GC-HBOC, a Familial Breast and Ovarian Cancer Center was implemented at the University Medicine Greifswald over a four-year period from 2018 to 2021, despite the COVID-19 pandemic. Genetic analyses were performed in a total of 658 individuals, including 41 males, which paved the way to local annual risk-adapted breast cancer surveillance for 91 women and prophylactic surgery for 34 women in 2021. Our experience in the North Eastern part of Germany demonstrates that it is possible to establish a high-risk breast and ovarian cancer service even in a sparsely populated region. Major facilitators are the interdisciplinary collaboration of dedicated local experts, the support of the GC-HBOC, fruitful clinical and scientific cooperations and the use of technical improvements. As a blueprint, our project report may help to further expand the network of specialized and knowledge-generating care for HBOC families.

## 1. Introduction

**Figure 1:**
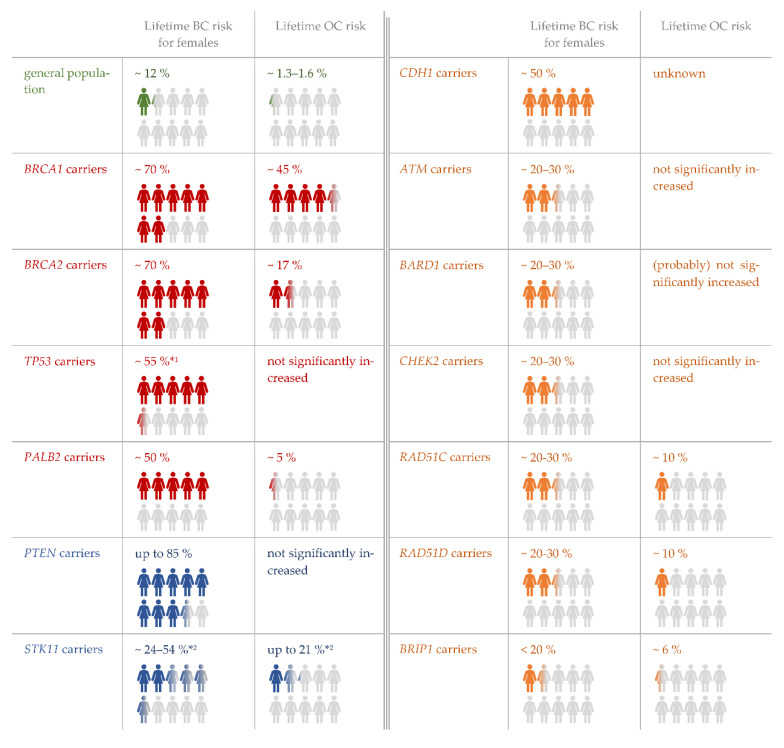
HBOC core genes and associated lifetime risks for breast and ovarian cancer in female carriers of pathogenic germline variants as reviewed in detail in the latest consensus recommendations of the GC-HBOC [1,3]. This illustration was designed for genetic counseling at the University Medicine Greifswald. High-risk genes are marked in red. Moderately penetrant genes are marked in orange. The syndrome-associated genes *PTEN* and *STK11* are marked in blue. *^1^ Lifetime breast cancer risk for females from families with Li-Fraumeni syndrome, *^2^ Lifetime risks for breast cancer and ovarian cancer (often sex cord tumor with annular tubules) in Peutz-Jeghers syndrome patients according to [4,5,6,7].

The identification of a germline variant in one of the core disease genes defined by the German Consortium Hereditary Breast and Ovarian Cancer (GC-HBOC) increases significantly the lifetime risk to develop breast and ovarian cancer (Figure 1). It also influences the medical management, determines the inclusion into a structured surveillance program and, depending on the genetic results, may give access to prophylactic surgery. Based on expert opinion and individual risk–benefit analyses, the GC-HBOC has recently updated its consensus recommendations for the nationwide clinical management of individuals carrying a pathogenic or likely pathogenic variant in one of the core genes [1]. These recommendations also take into account the different risk profiles of the core genes. Thus, high breast and, in some cases, ovarian cancer risks are observed in carriers of pathogenic variants of *BRCA1*, *BRAC2*, *PALB2* and *TP53*. Therefore, these genes are referred to as high-risk genes. Other genes, such as *CHEK2* or *ATM*, are classified as moderate–penetrance due to a lower lifetime cancer risk associated with them (Figure 1).

**Figure 2:**
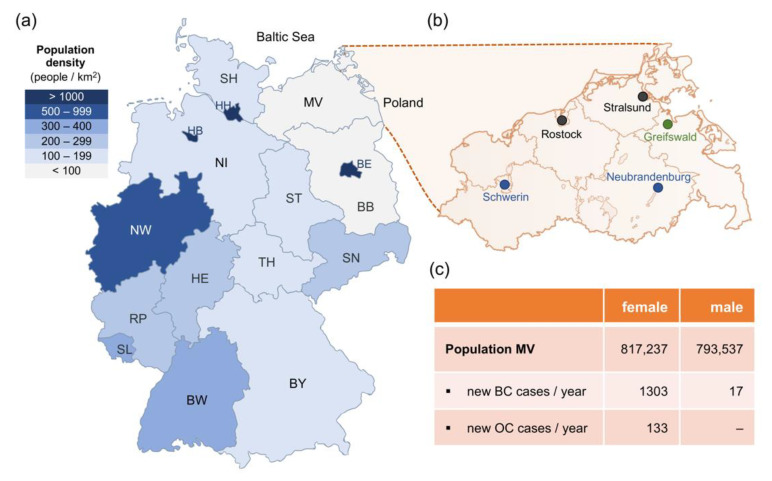
Geographical location of the Familial Breast and Ovarian Cancer Center Greifswald. (**a**) Population density in the 16 federal states of Germany in people per square kilometer (people/km^2^) [12], (**b**) Mecklenburg-Western Pomerania (MV) with its five largest cities. The Familial Breast and Ovarian Cancer Center Greifswald and its cooperation partners are marked in green and blue, respectively. Attribution for the map [13]. (**c**) Population and BC/OC statistics for Mecklenburg-Western Pomerania [10]. BW, Baden-Württemberg; BY—Bavaria; BE—Berlin; BB—Brandenburg; HB—Bremen; HH—Hamburg; HE—Hessen; MV—Mecklenburg-Western Pomerania; NI—Lower Saxony; NW—North Rhine-Westphalia; RP—Rhineland-Palatinate; SL—Saarland; SN—Saxony; ST—Saxony-Anhalt; SH—Schleswig-Holstein; TH—Thuringia.

Until 2018, there was no Familial Breast and Ovarian Cancer Center in the North Eastern part of Germany. To establish regional integrated care with the best possible quality for affected individuals and their healthy at-risk family members, a specialized center was established at the University Medicine Greifswald according to the standard operating procedures defined by the GC-HBOC. The organization of interdisciplinary expert-level human genetics, gynecological and radiological care in sparsely populated areas is challenging though. Only five hospitals currently meet the criteria to run certified breast cancer centers (BCC) within the entire German federal state Mecklenburg-Western Pomerania (Mecklenburg-Vorpommern, MV, Figure 2a,b), which are (1) the Interdisciplinary BCC at the University Medicine Greifswald, (2) the BCC at the Helios Hospital Schwerin, (3) the BCC at the Helios Hanse Hospital Stralsund, (4) the BCC at the Dietrich-Bonhoeffer Medical Center Neubrandenburg and (5) the University BCC at the Südstadt Hospital Rostock [2]. In 2018, the University Medicine Greifswald was the only institution that also provided an academic department of human genetics and a certified gynecological tumor center.

Unique selling points of the University Medicine Greifswald are the Study of Health in Pomerania (SHIP), which includes whole-body magnetic resonance imaging (MRI) [8] and a German-Polish cross-border teleradiology network funded by the European Union Interreg program in 2002 [9]. It covers the historic area of Pomerania and was implemented prior to the COVID-19 pandemic for two reasons: telemedicine enlarges the catchment area and delivers specialized medical services into a region that has the lowest population density in Germany and is flanked by the Baltic Sea in the north and the border to Poland in the east (Figure 2a).

For Mecklenburg-Western Pomerania, the joint cancer registry documented an average of 1303 new female and 17 new male breast cancer cases per year (Figure 2c). In addition, 133 new ovarian cancer cases were reported annually [10]. On the assumption that 30% of individuals affected with breast cancer meet the GC-HBOC inclusion criteria [11], it can be estimated that 391 individuals with breast cancer are eligible to receive genetic counseling and diagnostic genetic analyses of all consented HBOC core genes annually. Notably, 103 women affected by ovarian cancer were below the age of 80 years at diagnosis [10] and, thus, would also qualify for genetic counseling and analyses. This estimation does not yet include predictive genetic analyses, the increasing amount of therapeutic indications and individuals affected with triple-negative breast cancer (TNBC) between 50 and 59 years of age.

In 2018, major health insurance companies concluded contracts with the University Medicine Greifswald to cover the costs for the identification and surveillance of individuals with hereditary breast and ovarian cancer, including their at-risk relatives. Two years later, cooperations were started with the certified breast cancer center and the certified gynecological tumor center in MV’s capital city Schwerin and the certified breast cancer center at the Dietrich-Bonhoeffer Medical Center Neubrandenburg (Figure 2b). This project report describes the implementation and growth of the first prevention program for a genetic risk collective in the North Eastern part of Germany and illustrates the medical demands of patients and healthy individuals at risk of a familial tumor predisposition syndrome.

## 2. Materials and Methods

We here conducted a retrospective analysis of 658 patients with fulfilled inclusion criteria of the GC-HBOC, who were either screened for pathogenic germline variants in HBOC disease genes with the TruRisk gene panel or tested for a familial mutation at the Familial Breast and Ovarian Cancer Center Greifswald between January 2018 and December 2021. Additionally, 26 TNBC cases analyzed at the University Medicine Greifswald MVZ GmbH in accordance with the AGO guidelines [14] were reviewed. However, probes that were transferred to the University Medicine Greifswald MVZ GmbH for therapeutic *BRCA1*/*BRCA2* testing were excluded.

### 2.1. Implementation of the Clinical Framework

The standard operating procedures of the GC-HBOC were adjusted to the local conditions, and quality assurance measures were established, including accreditation of the molecular genetics laboratory according to DIN EN ISO 15189:2014 in 2018, certification of the Familial Breast and Ovarian Cancer Center according to ISO 9001:2015 in 2021, annual audits by an external company, interdisciplinary quality circles, training of the participating staff and integration of all documents into the quality management software roXtra.

### 2.2. Molecular Genetic Analyses

All gene panel, Sanger sequencing, and multiplex ligation-dependent probe amplification (MLPA) analyses were performed with written informed consent according to the German Gene Diagnostics Act. The NucleoSpin Blood L Kit (Macherey-Nagel, Düren, Germany) was used to isolate genomic DNA. The TruRisk v2 gene panel, which we had been validated with the support of the GC-HBOC, was used for hybridization capture-based next-generation sequencing (NGS) of the target genes: *ATM* (reference sequence: LRG_135t1, MIM: 607585), *BARD1* (LRG_297t1, 601593), *BRCA1* (LRG_292t1, 113705), *BRCA2* (LRG_293t1, 600185), *BRIP1* (LRG_300t1, 605882), *CDH1* (LRG_301t1, 192090), *CHEK2* (LRG_302t1, 604373), *PALB2* (LRG_308t1, 610355), *RAD51C* (LRG_314t1, 602774), *RAD51D* (LRG_516t1, 602954), and *TP53* (LRG_321t1 and LRG_321t2, 191170). *BRIP1* and *BARD1* were included as HBOC core genes in 2018 and 2019. *PTEN* (LRG_311t1, 601728), and *STK11* (LRG_319t1, 602216) were analyzed for patients with additional signs of PTEN hamartoma tumor syndrome (PHTS) or Peutz-Jeghers syndrome (PJS), respectively. For patients with OC or with a positive family history of hereditary nonpolyposis colorectal cancer (HNPCC)-related tumors, the target region was extended to *MLH1* (LRG_216t1, 120436), *MSH2* (LRG_218t1, 609309), *MSH6* (LRG_219t1, 600678), *PMS2* (LRG_161t1, 600259) and *EPCAM* (LRG_215t1, 185535, CNV analysis only). The Agilent SureSelect^QXT^ enrichment kit was used for target enrichment and library preparation (Agilent Technologies, Santa Clara, CA, USA). The indexed library was sequenced with 2  ×  150  bp paired-read runs on a MiSeq instrument (Illumina, San Diego, CA, USA). FASTQ files were generated with the MiSeq Reporter Software (Illumina). The SeqNext module of the Sequence Pilot software (JSI Medical Systems, Ettenheim, Germany) was used for read mapping, alignment, variant calling and copy number variation (CNV) analyses. Pathogenic or likely pathogenic variants were confirmed on a second DNA sample using Sanger sequencing or MLPA (MRC Holland, Amsterdam, The Netherlands). For all OC patients, the exons 11 to 15 of the *PMS2* gene were analyzed by Sanger sequencing after long-range PCR amplification [15]. A specific *PMS2* MLPA was also performed for these patients.

### 2.3. Establishment of a PCR Validation Assay for a RAD51D Deletion

The *RAD51D* deletion c.896_*505delinsT that we had identified by NGS gene panel sequencing in the family described in Section 3.3 was first verified in a second DNA sample by MLPA. Because MLPA analyses are labor-intensive and can be quite expensive if the ratio of diagnostic to control samples is unfavorable, we next established a specific PCR assay for the detection of the deletion. Two independent primer sets with binding sites upstream and downstream of the breakpoints were designed (F1: GGAGCAGGAGCATCAGGC; R1: TGTTCTTTGGGCCTCACTGG; F2: GGAGGCTCAAACCTGCCC; R2: AAGGGGAAATCAGGTGGCTC). The PCR products were separated by agarose gel electrophoresis. While only one band of the expected size was detected in healthy control subjects, we observed two bands in the index patient, which could be clearly assigned to the wild-type and the deletion allele, respectively. This assay allowed the rapid and cost-effective testing of additional family members.

### 2.4. Data Analysis and Visualization

The GraphPad Prism software (v.9.3.1, GraphPad Software, LA Jolla, CA, USA) was used to visualize the spectrum of identified pathogenic variants as a stacked bar chart. No statistical comparisons were performed in the present study.

## 3. Results

### 3.1. Familial Breast and Ovarian Cancer Center Greifswald

**Figure 3:**
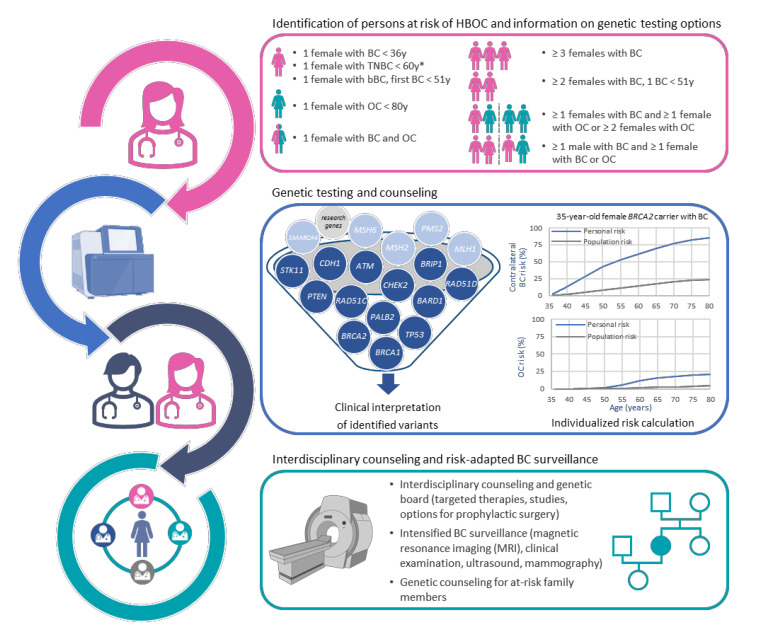
Integrated care for HBOC patients by an interdisciplinary team of experts at specialized Familial Breast and Ovarian Cancer Centers. * Genetic testing of individuals with triple-negative breast cancer (TNBC) was extended to the 50-to-59 age group in 2022. Created with BioRender.com (accessed on 21 September 2022).

The complexity and the amount of interdisciplinary care in any of the 23 Familial Breast and Ovarian Cancer Centers are summarized in Figure 3 and have been described in detail [1,16,17,18]. In these specialized centers, genetic diagnostics and clinical care are based on the best available evidence but also integrated into a knowledge-generating health care concept [19]. Thus, clinical and genetic data are collected in the HerediCaRe database to fill knowledge gaps, e.g., regarding the age-dependent penetrance of new risk genes. Furthermore, new inclusion criteria for genetic testing, which still have wider confidence intervals for the mutation detection rates and therefore have not yet been included into the EBM catalog which regulates the reimbursement of medical services in the outpatient sector in Germany, are further validated in larger cohorts in the GC-HBOC. Finally, interdisciplinary care is also flanked by GC-HBOC research projects that create new knowledge, e.g., in the areas of genetic diagnostics and clinical prevention. Ethical, legal and social implications, the development of new concepts of care and the dissemination of knowledge through health care education programs are also addressed within the GC-HBOC [19].

At the University Medicine Greifswald, the institute of human genetics is the first point of contact for those seeking advice. Trained employees operate a service hotline, evaluate the inclusion criteria according to a standardized check list [11,20] and take care of the appointment management. During the first genetic consultation, documented clinical findings are carefully evaluated, and the pedigree over at least three generations is electronically finalized. Furthermore, detailed information about the genetic laboratory examinations according to the German Genetic Diagnostics Act is provided.

With informed consent, multigene analyses with the TruRisk gene panel of the GC-HBOC are performed and evaluated according to the criteria published by the GC-HBOC [21,22] and the ACMG/AMP variant classification guidelines [23] in the accredited molecular genetics laboratory located within the institute of human genetics (details in Section 3.2). Based on the results of the molecular genetic analyses, the age of disease manifestation, the individual family history, the tumor biology and other non-genetic factors, the individual risk of female BC patients to develop contralateral breast cancer and ovarian cancer is calculated using the latest BOADICEA risk calculation model (Breast and Ovarian Analysis of Disease Incidence and Carrier Estimation Algorithm) as part of the CanRisk web tool [24,25,26]. This personalized risk assessment for foreseeable periods of time such as the 10-year breast cancer risk is a major topic during the second interdisciplinary counseling [16].

According to their individual risk situations, affected individuals as well as healthy relatives with a genetic risk receive one of three intensified breast cancer surveillance programs operated by specialized gynecologists and radiologists [16,27]. Organized by the institute of human genetics, individuals with non-standard findings such as rare missense variants leading to an attenuated tumor predisposition can also be presented to an interdisciplinary genetic board in which experts from gynecology, oncology, radiology, human genetics and pathology discuss, for example, study inclusion, targeted therapies or risk-reducing prophylactic interventions. Additionally, optional psycho-oncological counseling is offered, and contact to self-help groups (e.g., BRCA-network) established.

**Figure 4:**
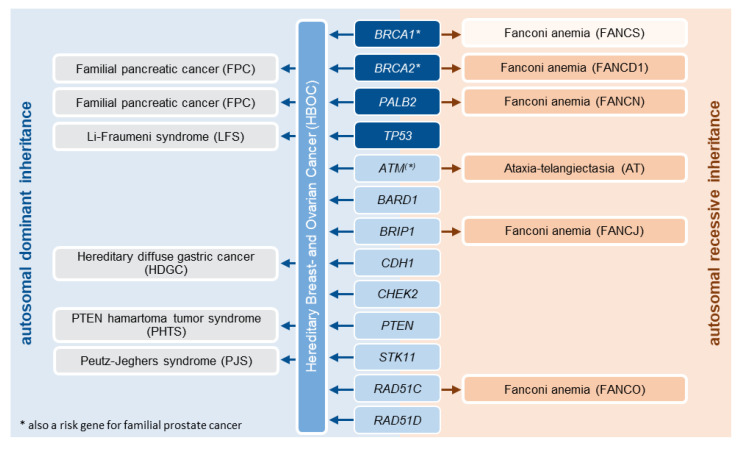
The simplified illustration of the HBOC core genes risk profile during genetic counseling at the institute of human genetics Greifswald does not claim completeness and does not replace a human genetics and interdisciplinary judgement, which depends on the individual genetic variant identified and is presented in the counseling letter and—if necessary—in the genetic board decision. Particularly, additional associated tumor risks (e.g., for *CHEK2* carriers [28]) are discussed in detail during the second genetic consultation.

Importantly, genetic counseling for at-risk family members may extend far beyond hereditary breast and ovarian cancer depending on the genetic variant identified. Thus, individuals with a pathogenic *BRCA2* or *PALB2* variant also have a significantly increased risk for pancreatic cancer. Increased risks for non-BC/OC tumors should also be considered for other genes, particularly *TP53*, *PTEN*, *STK11* or *CHEK2*. Furthermore, the offspring of individuals with a pathogenic germline variant in a specific risk gene may be at increased risk for autosomal recessive diseases. Homozygous or compound heterozygous *ATM* mutations, for example, cause ataxia telangiectasia. Likewise, biallelic *BRCA1*, *BRCA2*, *BRIP1*, *PALB2* and *RAD51C* mutations lead to Fanconi anemia (Figure 4).

### 3.2. Clinical Genetic Testing

#### 3.2.1. Individuals with Fulfilled Inclusion Criteria

Clinical genetic testing for individuals with a suspicious personal and/or family history is an essential component of care in Familial Breast and Ovarian Cancer Centers. To facilitate comprehensive and up-to-date diagnostics, the GC-HBOC developed the TruRisk gene panel in 2015, which is now regularly updated by an expert group. Apart from genes well known to be associated with increased breast and ovarian cancer risks (Figure 3), it also includes research genes identified in international collaborative studies [1]. Based on positive results of validation analyses for these candidate genes within the GC-HBOC, *BARD1* and *BRIP1* could be established as new diagnostic core genes in recent years [29,30]. *NBN*, on the other hand, was excluded from this group, because an association with increased breast cancer risks could not be confirmed [31].

After testing and validating the TruRisk panel, we performed 474 gene panel analyses from 2018 to 2021. Among these, there were 49 analyses for healthy counselees who can be tested if they come from families in whom no index patient with BC or OC is available and if the *BRCA1/2* gene mutation probability is ≥10% [18]. Starting with a moderate number of cases in the first year, we saw a continuous increase of 20 to 30% per year over the last four years, resulting in 173 complete gene panel analyses in 2021. Thus, the certification requirements of 150 complete gene panel analyses per year were met.

**Figure 5:**
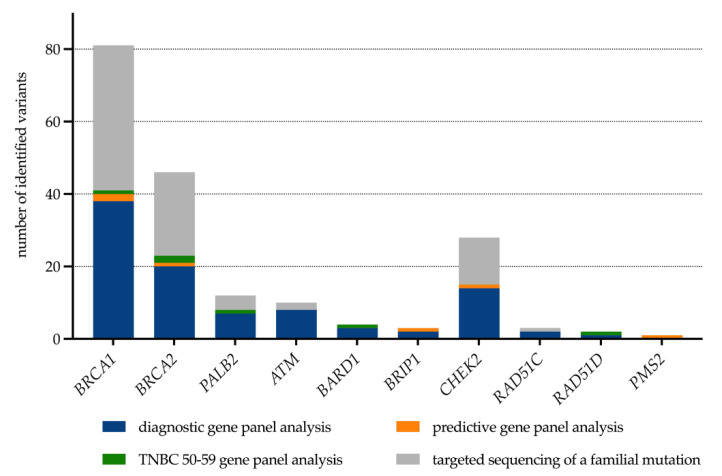
Spectrum of pathogenic or likely pathogenic variants identified in the establishing phase of the Familial Breast and Ovarian Cancer Center Greifswald. Of note, three patients carry mutations in two HBOC-associated risk genes.

A pathogenic germline variant in a high- or moderate-risk HBOC gene was identified in 98 cases (Figure 5). In three of them, we identified two pathogenic variants (*BRCA1*/*PALB2*, *BRCA1*/*ATM* and *PALB2*/*CHEK2*). If we exclude the predictive gene panel analyses from our dataset, a disease-causing variant was identified in 21.6% of all index patients with fulfilled GC-HBOC inclusion criteria. It is notable, however, that in our group of 49 healthy counselees who qualified for gene panel sequencing, we also observed a mutation detection rate of 12%. Two *BRCA1*, and one *BRCA2*, *CHEK2*, *BRIP1* and *PMS2* pathogenic variants were identified.

While the total mutation detection rate is comparable to previous GC-HBOC data [31,32], we observed a slight shift to pathogenic variants in moderate-penetrance risk genes. For example, we identified pathogenic *CHEK2* variants in more than 3% of cases that fulfilled the GC-HBOC criteria for genetic testing. This shift, however, was also accompanied by a change in our patient population, reflecting the ongoing refinement and expansion of inclusion criteria for clinical genetic testing. In 2017 and 2018, the consortium reported mutation detection rates of >10% for OC and TNBC patients with a negative family history who were diagnosed before the age of 80 or 50 years, respectively [33,34]. Since 10% is the threshold defined by international guidelines and accepted for reimbursement by German health insurance companies [19,33], the inclusion criteria could be extended to both groups. Most recent GC-HBOC data also demonstrated a mutation prevalence of >10% in male breast cancer patients with a negative family history [35]. Although the spectrum of the identified mutations may change due to these additional indications, it is important that more high-risk patients can now be offered genetic counseling and gene panel sequencing in Familial Breast and Ovarian Cancer Centers.

#### 3.2.2. Patients with TNBC in the 50-to-59 Age Group

The extension of the inclusion criteria to patients with TNBC below 60 years of age introduced at the end of 2021 is very important. Until that time, we detected six pathogenic or likely pathogenic variants in *BRCA1*, *BRCA2* (two), *PALB2*, *RAD51D* and *BARD1* among 26 patients with TNBC in the 50-to-59 age group. These patients had been transferred to the University Medicine Greifswald MVZ GmbH according to the AGO guidelines [14]. In our small cohort, we thus reached a mutation detection rate of 23%. Larger studies will have to show whether this criterion should be extended to TNBC in patients above 60 years of age.

#### 3.2.3. Variants of Unknown Significance (VUS)

Together with clinical and family data, all variants identified in the gene panel analyses were uploaded into the HerediCaRe database of the GC-HBOC [36]. In 21% of our cases, a VUS was identified. VUS should not lead to clinical recommendations but may cause uncertainty or even anxiety in some patients. For these variants, a recall is guaranteed by the GC-HBOC. An expert panel, the VUS Task Force [22], re-evaluates the VUS and reports changes in classification back to the local centers. Within the GC-HBOC research project HerediVar, which is funded by the German Cancer Aid, this re-evaluation will be automated and thus even faster in the future [21]. This is also important for the relatives of an index person, since predictive analyses can only be offered for (likely) pathogenic variants but not for VUS.

#### 3.2.4. Predictive and Diagnostic Genetic Analyses for At-Risk Family Members

In addition to 474 gene panel analyses performed in our center between 2018 and 2021, 184 individuals were tested for a familial pathogenic variant. A hereditary cancer predisposition syndrome was confirmed in 84 of these cases (=45.7%). Most analyses were performed in families in which a pathogenic *BRCA1* or *BRCA2* variant had been detected before.

Taken together, a total of 658 individuals, including 41 males, were tested in the Familial Breast and Ovarian Cancer Center Greifswald in the first four years of the establishing phase. These analyses paved the way to intensified breast cancer surveillance for 91 women and prophylactic surgery for 34 women in 2021.

### 3.3. Selected Family Report: Pathogenic BRCA2 and RAD51D Variants in an HBOC Family

**Figure 6:**
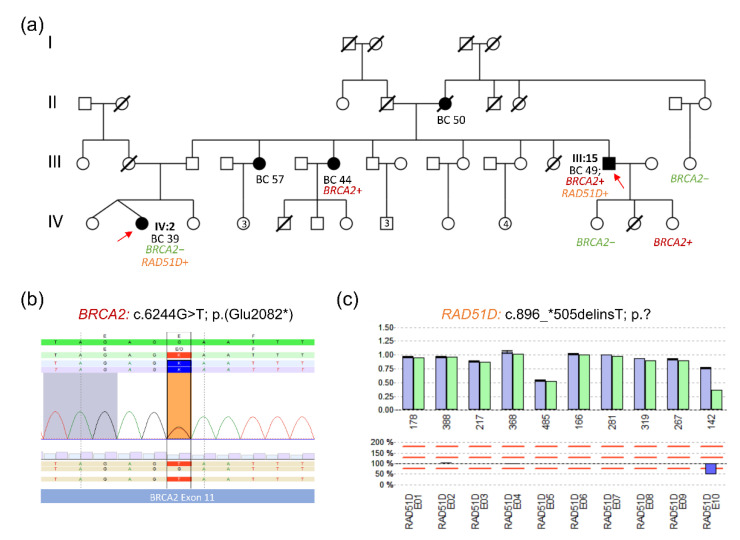
Identification of two different pathogenic germline variants in an HBOC family. (**a**) Pedigree of a family with carriers of pathogenic *BRCA2* and *RAD51D* variants. I-IV indicate the different generations of the family. (**b**) NGS data showing the variant in *BRCA2*: c.6244G > T; p.(Glu2082*). (**c**) Multiplex ligation-dependent probe amplification (MLPA) data showing a large deletion in *RAD51D*. Review of the NGS data specified heterozygosity for the *RAD51D* variant c.896_*505delinsT.

The introduction of NGS gene panel analyses into routine diagnostics in the last decade was quite a game changer and now gives us a much more comprehensive picture of genetic risks in HBOC families. Over the last few years, however, we have also learned that the individual situation may be much more complex than initially thought. As an example, one family is shown here (Figure 6).

The male index case III/15, who was diagnosed with multifocal, hormone receptor-positive, HER2-negative breast cancer at the age of 49 years, was tested by our laboratory in 2015 prior to our integration into the GC-HBOC. Sequence analysis for *BRCA1*, *BRCA2* and the most common *CHEK2* mutation c.1100delC identified a heterozygous *BRAC2* nonsense variant [c.6244G > T; p.(Glu2082*)] which was classified as pathogenic. In the following years, the *BRCA2* variant was also detected in one of his sisters, who developed breast cancer at age 44, and in one of his healthy daughters. In his oldest daughter and a maternal cousin, on the other hand, the variant could be excluded by predictive analyses.

Recently, the niece (IV/2) of the index patient developed TNBC at the age of 39 and presented at our Familial Breast and Ovarian Cancer Center. A targeted genetic analysis did not detect the known familial *BRCA2* variant. Since the niece herself fulfilled the GC-HBOC inclusion criteria, a complete gene panel analysis was performed as a second step. NGS-based copy number variation (CNV) analysis identified a heterozygous deletion in *RAD51D* which was verified by multiplex ligation-dependent probe amplification (MLPA). Re-evaluation of the NGS raw data confirmed that the breakpoints were located in exon 9 and the 3’ UTR (untranslated region) of *RAD51D* (c.896_*505delinsT). The deletion was listed in the HerediCaRe database and was classified as pathogenic by the VUS Task Force based on cDNA analysis in the GC-HBOC. Notably, a recently published study of the Breast Cancer Association Consortium (BCAC) with more than 60,000 female BC patients and more than 53,000 controls has demonstrated that protein truncation *RAD51D* variants are associated with a moderately increased BC risk [37]. Thus, intensified breast cancer surveillance will be offered to the patient starting 6 to 12 months after the completion of her current therapy. In addition, the indication for risk-reducing salpingo-oophorectomie upon entry into menopause has been discussed in accordance with the consensus recommendations of the GC-HBOC [1].

Finally, a specific PCR assay for the familial *RAD51D* deletion was designed to save the costs of the MLPA analyses, and the variant was also found in the male index case III/15. Supplementary predictive analyses can now be offered to his daughters and further relatives.

## 4. Discussion

Over the last decades, we have learned much about the genetics of familial breast and ovarian cancer, gene-specific cancer risk profiles and the benefits of intensified surveillance with MRI for high-risk patients [27,37]. However, a key to the successful clinical translation of this knowledge is the development of a nationwide infrastructure of specialized local centers where interdisciplinary care, genetic counseling and diagnostics and risk-adapted prevention come together in a knowledge-generating health care concept. This is one of the primary goals of the GC-HBOC [19]. The establishment of the Familial Breast and Ovarian Cancer Center Greifswald, which began in 2018, has closed a gap in the North Eastern part of Germany.

**Figure 7:**
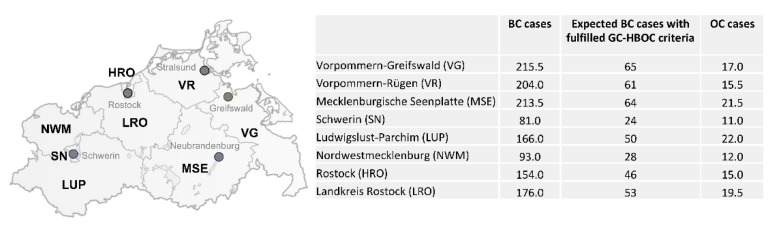
BC and OC cases in the districts of Mecklenburg-Western Pomerania. Shown are the average case numbers per year [10]. “Expected BC cases with fulfilled GC-HBOC criteria” are calculated assuming that 30% of them have a suspicious personal and/or family history [11]. Attribution for the map: [13].

Our data demonstrate that almost 35 to 40% of BC and OC patients in Mecklenburg-Western Pomerania who are expected to fulfill the GC-HBOC inclusion criteria are currently using the services offered by the new center in Greifswald (Figure 7). The steady increase in counselees, resulting in 173 gene panel tests and more than 70 analyses for a familial mutation in 2021, demonstrates a need for specialized care in the North Eastern part of Germany. Together with the two partner hospitals, the Helios Hospital Schwerin and the Dietrich-Bonhoeffer Medical Center Neubrandenburg, interdisciplinary care is offered to an area stretching from the two islands, Usedom and Rügen, into northern Brandenburg. Thanks to the close cooperation with the medical care center of the University Medicine Greifswald, patients whose insurance companies have not yet signed a contract with the center in Greifswald can also be offered regional genetic counseling and diagnostics. This service is essential in a sparsely populated state where it takes several hours to reach the nearest metropolitan areas. Considering the certification requirements, however, it is always a challenge to find a balance between the shortest possible travel distances for patients on the one hand and the accessible patient population in the health care area on the other. Indeed, in a more densely populated state, there would be at least two certified Familial Breast and Ovarian Cancer Centers in an area of comparable size. Therefore, reducing barriers to accessing specialized counseling and care in a rural state like Mecklenburg-Western Pomerania will remain a major challenge. Telemedical forms of care might help to connect medical experts and patients despite geographic separation in the future even better [38,39]. The COVID-19 pandemic has already led to a significant increase in the use of video conferencing systems. Especially in cases with negative genetic test results and no recommendation for intensified surveillance, videoconference counseling proved to be very useful.

The special care offered at the GC-HBOC Center Greifswald is a benefit for the patients, the participating experts and the local institutions. The interdisciplinary exchange in the newly established local genetic board, in which complex test results and risk calculations are discussed, is perceived as very positive by the participating experts from gynecology, human genetics, radiology and oncology. Furthermore, the opportunity to discuss variants of uncertain significance with the VUS Task Force, the exchange of ideas in the GC-HBOC working groups and the collaboration with colleagues from the other centers on internal quality assessments and scientific projects have also contributed to the successful establishing phase of our center and to the implementation of the latest research results into our diagnostics. An example is the establishment of the NGS-based CNV detection which has contributed to a significant reduction in the diagnostic turnaround time. As illustrated by our selected family report, this pipeline now also allows a robust CNV detection in moderate-penetrance risk genes without additional MLPA analyses.

With support of the GC-HBOC, high-quality integrated care for affected individuals and their healthy at-risk relatives is now offered to patients who previously had long travel distances to the next GC-HBOC centers and, therefore, often did not take advantage of specialized care. Technical improvements, extended inclusion criteria, dedicated employees with broad experience and interpersonal collaborations were key factors to the successful implementation of a Familial Breast and Ovarian Cancer Center in the North Eastern part of Germany. For the future success of the center, it will be crucial to recruit additional qualified staff, sign contracts with more health insurance companies, complete the certification process and find new ways to provide care in a sparsely populated region in order to reach more patients.

## Data Availability

Not applicable.
